# Changing Socioeconomic Indicators of Human Plague, New Mexico, USA

**DOI:** 10.3201/eid1807.120121

**Published:** 2012-07

**Authors:** Anna M. Schotthoefer, Rebecca J. Eisen, Kiersten J. Kugeler, Paul Ettestad, Pamela J. Reynolds, Ted Brown, Russell E. Enscore, James Cheek, Rudy Bueno, Joseph Targhetta, John A. Montenieri, Kenneth L. Gage

**Affiliations:** Centers for Disease Control and Prevention, Fort Collins, Colorado, USA (A.M. Schotthoefer, R.J. Eisen, K.J. Kugeler, R.E. Enscore, J.A. Montenieri, K.L. Gage);; New Mexico Department of Health, Santa Fe, New Mexico, USA (P. Ettestad, P.J. Reynolds [retired]);; New Mexico Environment Department, Santa Fe (T. Brown [retired]);; Indian Health Services, Albuquerque, New Mexico, USA (J. Cheek);; Harris County Public Health and Environmental Services, Houston, Texas, USA (R. Bueno, Jr.);; and City of Albuquerque Division of Environmental Health, Albuquerque (J. Targhetta)

**Keywords:** Yersinia pestis, plague, New Mexico, socioeconomic indicators, socioeconomic risk factors, US Census Bureau data, bacteria

## Abstract

Socioeconomic indicators associated with temporal changes in the distribution of human plague cases in New Mexico were investigated for 1976–2007. In the 1980s, cases were more likely in census block groups with poor housing conditions, but by the 2000s, cases were associated with affluent areas concentrated in the Santa Fe–Albuquerque region.

## Abstract 2

Plague, a rare but severe disease spread by rodents and fleas, has been traditionally associated with poor, unsanitary living conditions. To test this association, researchers in New Mexico used census data to determine the geographic and socioeconomic status of plague patients. Although they confirmed that most cases occurred in areas where the habitat supports rodents and fleas, they also found a surprising shift to more middle- to upper-class neighborhoods. In the 1980s, most cases occurred where housing conditions were poor. By the 2000s, cases were occurring in the affluent Santa Fe and Albuquerque regions. Although the cause of this shift is unknown, possibilities include relocation of affluent families to plague-prone areas or improved socioeconomic conditions among those already living in plague-prone areas.

Plague is a severe zoonotic disease caused by *Yersinia pestis*. An average of 11 cases per year have occurred in the United States (range 1–40 cases) since 1976 (*1*); most cases in recent decades have been found in New Mexico (*2**,**3*). The pathogen cycles between rodents and fleas, and humans most frequently become infected through flea bites (*4*). Living near habitats that support the sylvatic cycle is a major risk factor associated with human disease in New Mexico (*2**,**5**,**6*). However, even in areas defined as high risk on the basis of environmental characteristics (*6*), plague is rare, and the area defined as highly suitable for plague represents a large geographic region (≈52,626 km^2^).

Poor socioeconomic status has been anecdotally associated with human plague cases, but this factor has rarely been investigated quantitatively in the United States, and such information has not been systematically collected for cases of *Y. pestis* infection. Identifying human socioeconomic or behavioral risk factors may enable a more refined definition of the highest risk populations for more targeted control efforts. To evaluate possible associations between socioeconomic factors and plague risk, we used US Census Bureau decennial data to compare census block groups (CBGs) in which human plague cases occurred and did not occur in New Mexico during 1976–2007.

## The Study

We restricted our analyses to peridomestic cases that were reported in the geographic region previously determined to be at high risk on the basis of environmental factors (*6*). Thus, we included 123 (75.9%) of 162 cases reported in New Mexico during the study period. We also restricted our analyses to the CBGs that had population densities within the range of densities found in plague-positive CBGs (0.05 and 1,425.40 persons/km^2^). This restriction avoided comparing rural to urban CBGs because plague tends to occur in rural to lightly suburbanized areas. The final area considered encompassed ≈17% of New Mexico (*6*) and included the entirety or portions of 483 CBGs (Figure; Table 1).

**Figure:**
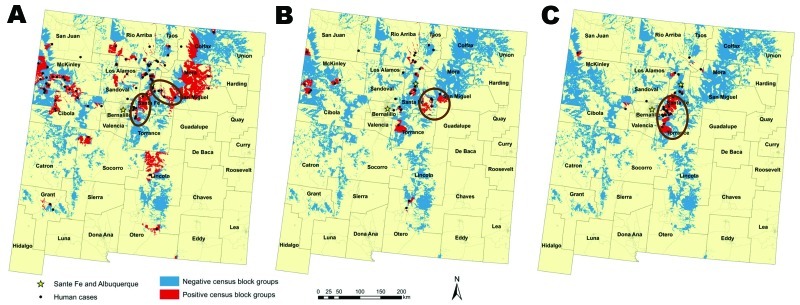
Areas of New Mexico, USA, considered in the current analysis on the basis of those defined as high risk for human plague by Eisen et al. (*6*) for each time frame examined. A) 1976–1985, B) 1986–1995, C) 1996–2007. Distributions of human cases are displayed and census block groups are color coded as negative or positive for plague cases. Census block group boundaries are indicated in light gray, and counties are outlined in dark gray. Ovals or circle indicate census block groups with significantly (p<0.05) high human plague incidence rates per 1,000 persons, identified by using the Kulldorff space scan statistic (*9*). Analyses were conducted by using the Poisson probability model and 999 Monte Carlo replications to test for significance.

**Table 1:** Characteristics of census block groups considered in analysis for human plague on the basis of 1980, 1990, and 2000 US Census data, New Mexico, USA*

Variable	1976–1985			1986–1995			1996–2007	
	Positive, n = 64	Negative, n = 405		Positive, n = 29	Negative, n = 430		Positive, n = 20	Negative, n = 434
Population density/km^2^	**4.34 (11.54)**	**22.68 (264.48)†**		8.31 (34.66)	23.46 (238.31)		13.70 (31.82)	25.96 (267.56)
Housing density/km^2^	**1.46 (4.18)**	**8.38 (107.56)†**		3.33 (12.57)	9.33 (95.91)		5.87 (12.13)	11.30 (107.09)
Poverty rate‡	**0.34 (0.23)**	**0.22 (0.23)§**		0.32 (0.42)	0.28 (0.23)		**0.16 (0.18)**	**0.24 (0.24)¶**
% Housing units								
Vacant	12.0 (8.84)	10.8 (7.09)		14.0 (15.54)	14.5 (16.34)		8.4 (10.77)	10.7 (18.46)
Rural farms	**2.1 (5.19)**	**1.3 (6.03)§**		0 (1.18)	0 (0.66)		0 (1.02)	0 (1.24)
Occupied, incomplete plumbing#	**8.6 (15.93)**	**2.5 (6.22)†**		**7.5 (30.57)**	**2.3 (9.21)§**		1.5 (5.77)	1.1 (4.47)
Occupied mobile homes	16.2 (5.89)	15.5 (17.88)		17.4 (23.31)	23.7 (20.25)		17.1 (13.19)	18.4 (24.21)
Built before 1940	**16.0 (14.98)**	**11.4 (16.92)§**		6.7 (16.81)	5.0 (15.67)		3.7 (10.38)	3.8 (11.72)
>40 y old	**16.0 (14.98)**	**11.4 (16.92)§**		14.3 (20.38)	11.1 (22.71)		8.3 (22.42)	15.8 (25.83)
<5 y old	20.6 (11.06)	21.7 (19.65)		17.1 (21.42)	13.6 (13.97)		21.5 (15.43)	14.5 (14.86)§
Heated with wood fuel	**18.5 (20.80)**	**6.8 (16.73)†**		**31.56 (34.65)**	**13.0 (31.99)†**		**16.9 (21.79)**	**6.8 (20.91)¶**
Occupied by >6 persons	**9.5 (6.28)**	**7.1 (4.89)§**		5.4 (11.97)	5.0 (6.90)		3.3 (4.48)	3.5 (5.94)
Household income**	**$28,477 ($11,544)**	**$30,190 ($16,367)¶**		$29,644 ($30,784)	$28,822 ($16,775)		**$44,098 ($30,563)**	**$31,323 ($18,541)§**
Value of homes**	$85,280 ($67,672)	$95,791 ($78,706)		$83,614 ($104,492)	$80,237 ($75,854)		**$132,350 ($90,050)**	**$84,300 ($90,400)¶**
Year housing unit built	NA	NA		1972 (9)	1974 (11)		**1987 (13)**	**1979 (11)§**
% Census block group area								
Ecotone habitat††	**2.8 (11.36)**	**0 (3.34)†**		**5.5 (13.44)**	**0 (4.33)†**		**10.7 (23.28)**	**0 (4.63)†**
Water	0.60 (0.45)	0.56 (0.83)		0.60 (0.31)	0.56 (0.71)		0.43 (0.33)	0.58 (0.67)

*Values are medians (interquartile ranges) for plague-positive and negative census block groups in the respective time frames. **Boldface** indicates variables significantly different between positive and negative groups. NA, not available. †p≤0.001, by Wilcoxon rank sum test. ‡Defined by the US Census Bureau as the proportion of the population living near or below the federal poverty line, which is based on household income adjusted for number of household members. The definition of the federal poverty line changed between the 1980 and 1990 censuses. Therefore, rates are not directly comparable across decennials. §p≤0.01, by Wilcoxon rank sum test. ¶p≤0.05 by Wilcoxon rank sum test. #Defined by the US Census Bureau as lacking >1 of the following: hot and cold piped water, a flush toilet, and a bathtub or shower. Values are not directly comparable among censuses. In 1980, a housing unit was considered to have complete plumbing only if plumbing fixtures were for exclusive for the residents of that unit. In 1990, the requirement of exclusive use was dropped. **In US year 2000–adjusted dollars, rounded to the nearest dollar. ††Identified as the convergence of the Rocky Mountain/Great Basin open and closed coniferous woodland habitats by Eisen et al. (*6*).

To relate plague occurrence to socioeconomic conditions of CBGs at times when cases occurred, we divided the study period into 3 time frames centered on the most recent census. Thus, 1976–1985 cases were associated with 1980 census data, 1986–1995 cases with 1990 census data, and 1996–2007 cases with 2000 census data. Variables that described economic status and housing conditions were extracted from each census, normalized to US Census 2000 CBG boundaries (Geolytics, Inc., East Brunswick, NJ, USA; www.geolytics.com), and compared between plague-positive and plague-negative CBGs by using Wilcoxon rank sum tests (Table 1). For each time frame, the risks of CBGs having at least 1 case of human plague on the basis of significant variables were then evaluated by using 2 × 2 tables. CBGs were divided into high and low categories by using median values of each variable as division points.

Plague risk was positively associated with CBGs that had an ecotone habitat identified by Eisen et al. (*6*) as especially suitable for human plague cases (e.g., convergence of the Rocky Mountain/Great Basin open and closed coniferous woodland habitats; odds ratio 4.18, 95% CI 2.66–6.57). Therefore, to ensure that we were measuring differences in socioeconomic conditions and not the presence or absence of the ecotone habitat in CBGs, we also calculated adjusted odds ratios for each variable and time frame by using Mantel-Haenszel tests.

Our results suggested temporal changes in socioeconomic factors associated with location of human plague cases. In the 1980s, plague tended to occur in CBGs with poor housing conditions (e.g., old homes with incomplete plumbing) and high proportions of the population living near or below the poverty line, but this second association was confounded by presence of ecotone habitat (Table 2; Figure). Beginning in the 1990s, plague cases began to be associated with CBGs with higher median incomes and home values, and by the 2000s, wealthier CBGs with higher proportions of newer homes were positively associated with plague cases (Table 1, Table 2). High proportions of homes using wood fuel were consistently associated with positive CBGs for each time frame (Table 1, Table 2), which supported suggestions from previous studies that availability of harborage for rodents (e.g., wood piles) in and around domestic environments may increase human plague risk (*2**,**5**–**8*).

**Table 2:** Socioeconomic indicators and human plague cases among CBGs, New Mexico, USA*

Indicator	1976–1985			1986–1995			1996–2007	
	% Positive	OR (95% CI)		% Positive	OR (95% CI)		% Positive	OR (95% CI)
Population in poverty†								
High	64.1	**1.81 (1.05–3.12)**		55.2	1.24 (0.58–2.65)		35.0	0.52 (0.21–1.34)
Low	35.9			44.8			65.0	
Value of homes								
High	40.6	0.64 (0.38–1.10)		51.7	1.07 (0.50–2.27)		75.0	**3.11 (1.11–8.71)‡**
Low	59.4			48.3			25.0	
Housing 0–5 y old								
High	48.4	0.93 (0.55–1.58)		65.5	1.97 (0.90–4.34)		75.0	**3.14 (1.12–8.79)‡**
Low	51.6			34.5			25.0	
Incomplete plumbing§								
High	84.4	**6.68 (3.31–13.49)‡**		65.5	1.97 (0.90–4.34)		55.0	1.23 (0.50–3.04)
Low	15.6			34.5			45.0	
Use of wood fuel								
High	84.4	**6.68 (3.31–13.49)‡**		79.3	**4.17 (1.66–10.44)‡**		75.0	3.14 (1.12–8.79)
Low	15.6			20.7			25.0	

*Boldface indicates significant associations (p<0.05). CBGs, census block groups; OR, odds ratio. †Defined by the US Census Bureau as the proportion of the population living near or below the federal poverty line, which is based on household income adjusted for number of household members. The definition of the federal poverty line changed between the 1980 and 1990 censuses, Therefore, rates are not directly comparable across decennials. ‡Variables that continued to be significant after controlling for presence of ecotone habitat. §Defined by the US Census Bureau as lacking >1 of the following: hot and cold piped water, a flush toilet, and a bathtub or shower. Values are not directly comparable among censuses. In 1980, a housing unit was considered to have complete plumbing only if plumbing fixtures were for exclusive for the residents of that unit. In 1990, the requirement of exclusive use was dropped.

A general change in the distribution of plague cases during the study period was also observed. In the 1980s, plague cases were more widely distributed across New Mexico and were particularly common in the northwestern region of McKinley and Cibola Counties (Figure). However, by the 1990s, plague cases became less common there and more focused in the north-central region of the state (Santa Fe–Albuquerque and surrounding counties; Figure). We implemented the Kulldorff spatial scan statistic (*9*) by using SaTScan (*10*) to identify clusters of CBGs with high incidence rates of plague cases per 1,000 persons for each of the time frames to quantify these changes. Significant clusters were detected only in the Santa Fe–Albuquerque region for each time frame (Figure). Changes consistent with the overall analysis in which plague occurrence shifted from poorer to wealthier CBGs and occurred in more new homes were observed when this region was considered alone.

Our analysis also suggested that migration of middle to upper–class families into suitable plague habitat throughout the high-risk areas of the state was associated with locations of plague cases. For example, in the 1990s, 28 (96.6%) of 29 plague-positive CBGs experienced population growth between the 1980 and 1990 censuses, in contrast to 337 (78.4%) of 430 nonplague CBGs that experienced growth. Likewise, 17 (85%) of 20 plague-positive CBGs in the 2000s occurred where there was growth between the 1990 and 2000 censuses versus growth in only 327 (75%) of 434 nonplague CBGs. Moreover, for the census 2000 period, population growth was more likely to have occurred in CBGs that had ecotone habitat than CBGs without ecotone habitat (p = 0.004, by Fisher exact test). Migration of persons into suitable plague habitat would potentially increase the likelihood of human exposure to infected rodents and their fleas (*7*).

## Conclusions

Overall, our results confirmed the role of living in or near habitats that support maintenance of sylvatic plague as a risk factor for human *Y. pestis* infection, but also suggested migration of middle to upper–class families into such areas may be contributing to changes in the locations of plague cases. The north-central region of New Mexico surrounding Santa Fe and Albuquerque was identified as a persistent focus of human plague cases, and it appears to be the predominate region for current cases. It is unclear why cases have become rare in the northwestern region of New Mexico because socioeconomic conditions have not generally improved there. However, the high numbers of cases observed there in the 1980s were associated with favorable climatic conditions for plague (*11*).

Although we detected changes in the socioeconomic indictors associated with the locations of plague-positive CBGs, what shifting individual behavioral factors may have accompanied these trends are unknown. In particular, we were unable to determine whether the socioeconomic status of individual plague case-patients has changed from poor to middle or upper–income classes. Future investigations are needed to characterize the characteristics and behaviors of persons to verify and fully understand the changing factors associated with plague cases in New Mexico.
